# The Musculature of Coleoid Cephalopod Arms and Tentacles

**DOI:** 10.3389/fcell.2016.00010

**Published:** 2016-02-18

**Authors:** William M. Kier

**Affiliations:** Department of Biology, University of North CarolinaChapel Hill, NC, USA

**Keywords:** arm, biomechanics, cephalopod, muscle, muscular-hydrostat, myosin, obliquely striated, tentacle

## Abstract

The regeneration of coleoid cephalopod arms and tentacles is a common occurrence, recognized since Aristotle. The complexity of the arrangement of the muscle and connective tissues of these appendages make them of great interest for research on regeneration. They lack rigid skeletal elements and consist of a three-dimensional array of muscle fibers, relying on a type of skeletal support system called a muscular hydrostat. Support and movement in the arms and tentacles depends on the fact that muscle tissue resists volume change. The basic principle of function is straightforward; because the volume of the appendage is essentially constant, a decrease in one dimension must result in an increase in another dimension. Since the muscle fibers are arranged in three mutually perpendicular directions, all three dimensions can be actively controlled and thus a remarkable diversity of movements and deformations can be produced. In the arms and tentacles of coleoids, three main muscle orientations are observed: (1) transverse muscle fibers arranged in planes perpendicular to the longitudinal axis; (2) longitudinal muscle fibers typically arranged in bundles parallel to the longitudinal axis; and (3) helical or obliquely arranged layers of muscle fibers, arranged in both right- and left-handed helixes. By selective activation of these muscle groups, elongation, shortening, bending, torsion and stiffening of the appendage can be produced. The predominant muscle fiber type is obliquely striated. Cross-striated fibers are found only in the transverse muscle mass of the prey capture tentacles of squid and cuttlefish. These fibers have unusually short myofilaments and sarcomeres, generating the high shortening velocity required for rapid elongation of the tentacles. It is likely that coleoid cephalopods use ultrastructural modifications rather than tissue-specific myosin isoforms to tune contraction velocities.

## Introduction

Regeneration in cephalopods has been recognized since Aristotle (Bello, 1995) and was initially observed in individuals collected with arms or tentacles in the process of regeneration (Verrill, 1881; Brock, 1886; Lange, 1920). Regeneration is common and occurs following injury to the arms and tentacles from predators (Duval et al., 1984) and in species capable of arm autotomy (Norman, 1992; Hochberg et al., 2006). Arm anomalies such as supernumerary development and arm agenesis have also been observed (Toll and Binger, 1991). Aspects of the regeneration of the arms and tentacles have been described (Lange, 1920; Adam, 1937; Aldrich and Aldrich, 1968; Féral, 1978, 1979, 1988; Fossati et al., 2013; Tressler et al., 2014) but the process is poorly understood at present. For a review of the current understanding of regeneration in cephalopod arms and tentacles please see the companion article by Zullo et al. (in review). When the regeneration process is complete, the new arm or tentacle is indistinguishable from the original (Tressler et al., 2014). As will be described below, the morphology and arrangement of the muscle and connective tissues is quite complex, with fibers running in precisely aligned and frequently interdigitated arrays. This complexity of structure is also true of the nervous system of the arms and tentacles. It is thus challenging and of great interest to understand the processes involved in regulating the regeneration of such complicated organs.

The goal of this paper is to provide an overview of the morphology of coleoid cephalopod arms and tentacles with a particular focus on the arrangement of the musculature and the biomechanics of movement and support. In addition, the ultrastructure of the muscle and its contractile properties will be described, along with information on the biochemistry of the myofilament lattice and the possible mechanisms of muscle fiber specialization.

## Muscle morphology and biomechanics

The arrangement of the musculature of the arms and tentacles and indeed that of the body of coleoid cephalopods is characterized by a three-dimensional array of muscle fibers (Kier, 1988; Zell, 1988; Kier and Thompson, 2003). These appendages lack the rigid internal or external skeletal elements that are present in the skeletons of vertebrates and arthropods and, in addition, they lack the fluid-filled cavities that characterize the hydrostatic skeleton of many invertebrates (Chapman, 1950, 1958, 1975; Clark, 1964, 1981; Wainwright, 1970, 1982; Gutmann, 1981; Kier, 2012). Support, transmission of force, muscular antagonism, and amplification of force or displacement are thus not provided by a conventional rigid skeleton or hydrostatic skeleton. Instead, as will be described below, the musculature itself serves both as the effector of movement and also as the skeletal support. In the descriptions below it should be kept in mind that the muscle cells of cephalopods are small, typically less than a millimeter in length (Bone et al., 1981; Milligan et al., 1997; Feinstein et al., 2011).

### Squid and cuttlefish tentacles

In squid and cuttlefish, one pair of the ten appendages surrounding the mouth, termed tentacles, is specialized for capturing prey. This behavior involves a remarkably rapid elongation of the tentacles, bringing the terminal clubs, which are equipped with suckers, into contact with the prey (Messenger, 1968, 1977; Kier, 1982). In loliginid squid the prey strike occurs in 20–40 ms and involves 40–80% elongation of the tentacles with maximum extension velocities of over 2 m s^−1^, and peak accelerations of approximately 250 m s^−2^ (Kier and van Leeuwen, 1997). The suckers attach to the prey and the tentacular stalks shorten, bringing the prey within reach of the eight arms, which then subdue and manipulate the prey for ingestion. Once the prey has been caught and transferred to the arms, the tentacles are released from the prey and are not involved further in prey handling.

#### Morphology and microanatomy of the tentacle musculature

**Figure 1:**
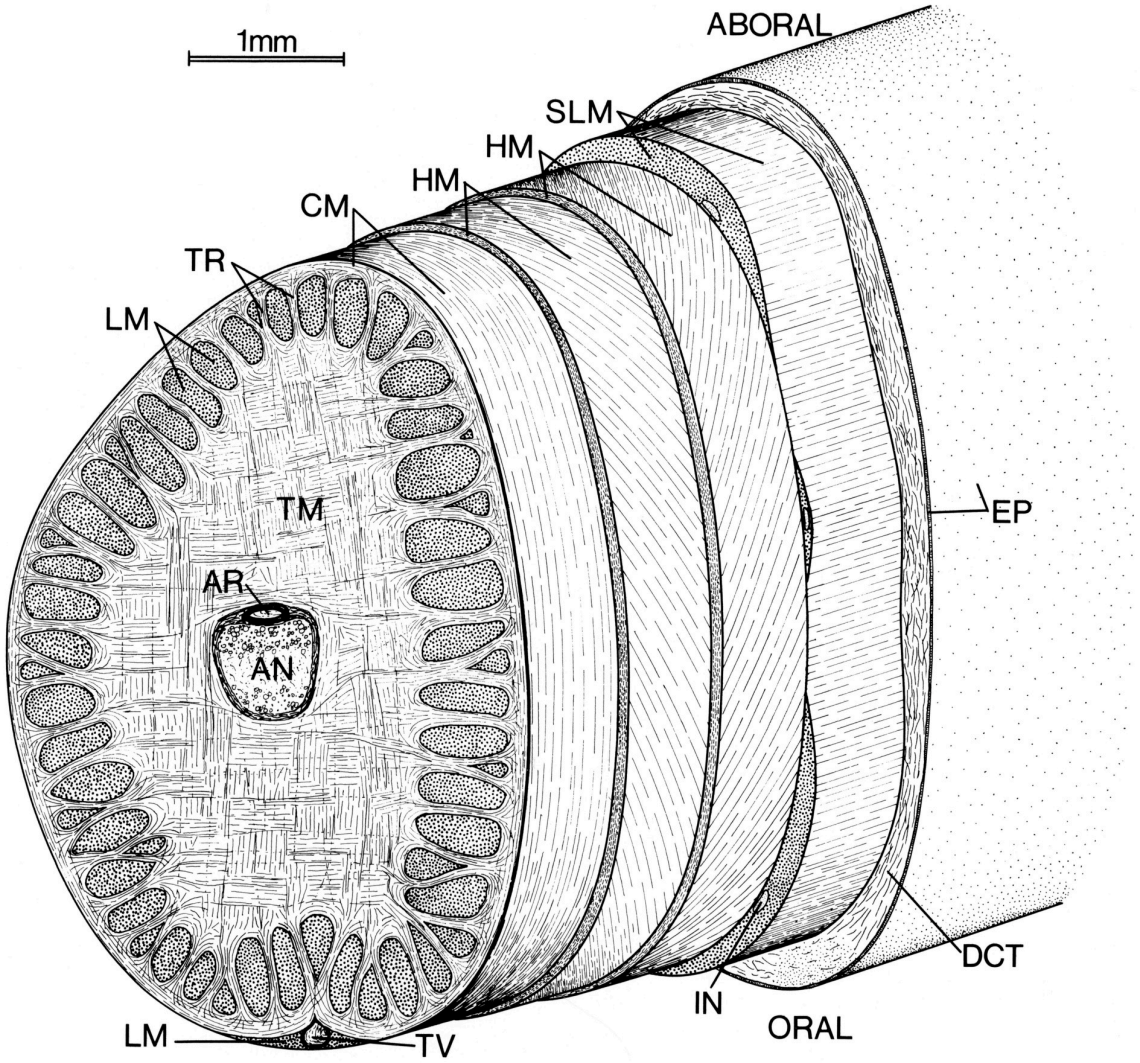
**Schematic diagram of left tentacular stalk of a loliginid squid**. AN, Axial nerve cord; AR, artery; CM, circular muscle; DCT, dermal connective tissue; EP, epithelium; HM, helical muscle; IN, intramuscular nerve cord; LM, longitudinal muscle; SLM, superficial longitudinal muscle; TR, trabeculae of transverse muscle; TM, transverse muscle; TV, superficial tentacular vein. From Kier (1982).

The axial nerve cord runs longitudinally down the center of the tentacle stalk and is surrounded by an extensive mass of transverse muscle (Figure 1; Guérin, 1908; Kier, 1982). Muscle fiber bundles in this mass extend across the diameter of the tentacle, perpendicular to its longitudinal axis. As the transverse muscle fiber bundles extend toward the periphery they pass in between bundles of longitudinal muscle fibers oriented parallel to the longitudinal axis of the tentacle. As they approach the external surface of the stalk, some of the fiber bundles from the transverse muscle mass can be observed to turn and become part of a thin circular muscle layer that surrounds the core of transverse and longitudinal muscle.

The circular muscle layer is wrapped by a pair of thin layers of helically oriented muscle fibers. The inner and outer layers are opposite in handedness and the fiber angle (the angle that the helical fibers make with the longitudinal axis) varies from a maximum of approximately 67° in a retracted tentacle to a minimum of approximately 36° in a fully extended tentacle for *Doryteuthis pealeii* (Kier, 1982). The helical muscle layers are surrounded by a layer of superficial longitudinal muscle.

Connective tissue is present in layers surrounding the axial nerve cord and at the interface between the various muscle groups described above. Connective tissue is also present between the muscle fibers in the various muscle masses. Surrounding the entire tentacular stalk is a layer of loose dermal connective tissue containing chromatophores, blood vessels, and nerves. A simple cuboidal to columnar epithelium covers the entire tentacular stalk.

#### Biomechanics of support and movement in the tentacles

Support and movement relies on the fact that the muscle and other tissues do not undergo significant change in volume in response to changes in pressure (Kier, 1982; Kier and Smith, 1985; Smith and Kier, 1989). The basic principle of function is straightforward. Since the volume of the tentacles is essentially constant, a decrease in one dimension must result in an increase in another dimension. Thus, the rapid elongation of the tentacles during the prey strike is caused by contraction of the transverse and associated circular muscle fibers; their shortening decreases the cross-sectional area and since there is insignificant decrease in volume, the length must increase. Shortening of the tentacles is caused by contraction of the longitudinal muscle, which increases the cross-section and thereby re-extends the transverse musculature. The transverse and longitudinal muscles thus serve as antagonists in a manner analogous to muscles on opposite sides of the joint of a vertebrate (Kier, 1982).

**Figure 2:**
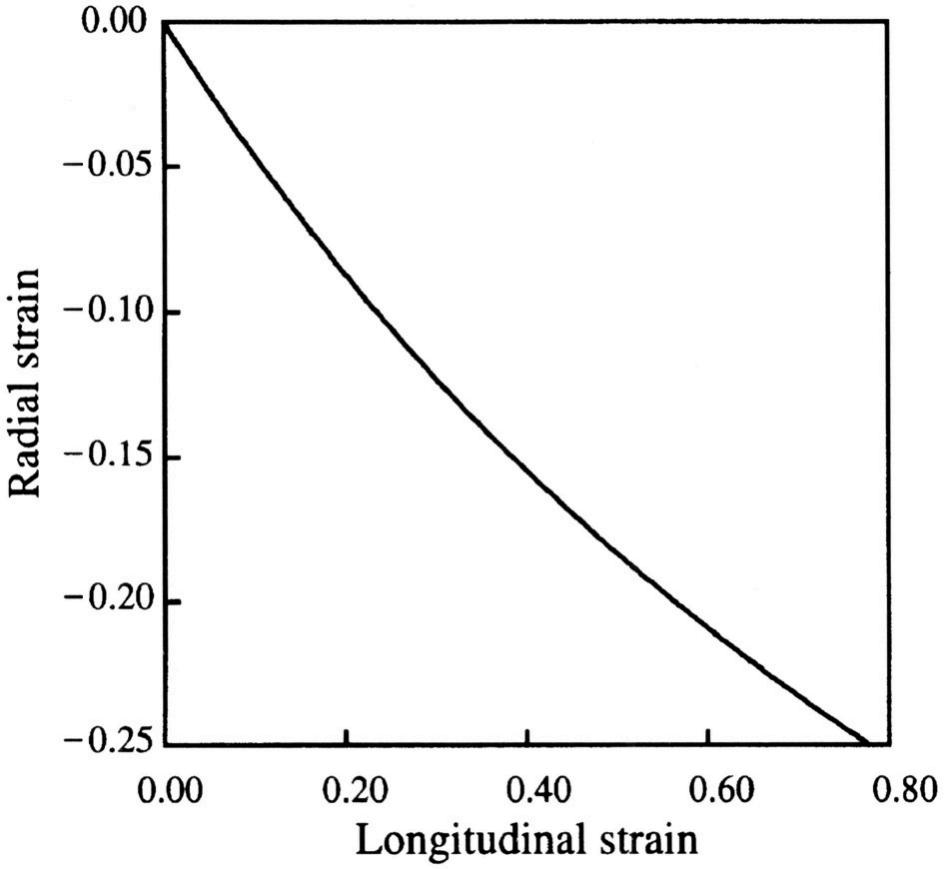
**Relationship between longitudinal and radial strain in the tentacle**. From van Leeuwen and Kier (1997).

The displacement and velocity of contraction of the transverse and circular muscle fibers is amplified. Because the transverse muscle is arranged in an orthogonal pattern, its contraction decreases both the width and the height of the tentacle. This decrease in cross-section represents a decrease in area (length squared) that results in tentacle elongation (length to the first power) and thus the shortening of the transverse and circular muscle is amplified. The relationship between the radial strain, ε_*r*_ and longitudinal strain, ε_*l*_ is

$$\begin{array}{l} \varepsilon_{r}={\left(1+\varepsilon_{l}\right)}^{-\frac{1}{2}}-1 \end{array}$$

(van Leeuwen and Kier, 1997). For instance, during an elongation of the tentacles of approximately 80%, a value typically observed during prey capture, a decrease in diameter of only 25% is required (Kier, 1982; van Leeuwen and Kier, 1997, 1998; Figure 2). This amplification of displacement is analogous to leverage in skeletons with rigid skeletal elements that have relatively shorter input than output arms. Mechanical amplification is in part responsible for the rapidity of the tentacle strike (van Leeuwen and Kier, 1997) and, in addition, the transverse and circular muscle fibers show specializations for high shortening velocity (see Section Mechanisms Responsible for Differences in Contractile Properties of Arm and Tentacle Transverse Muscle below).

**Figure 3:**
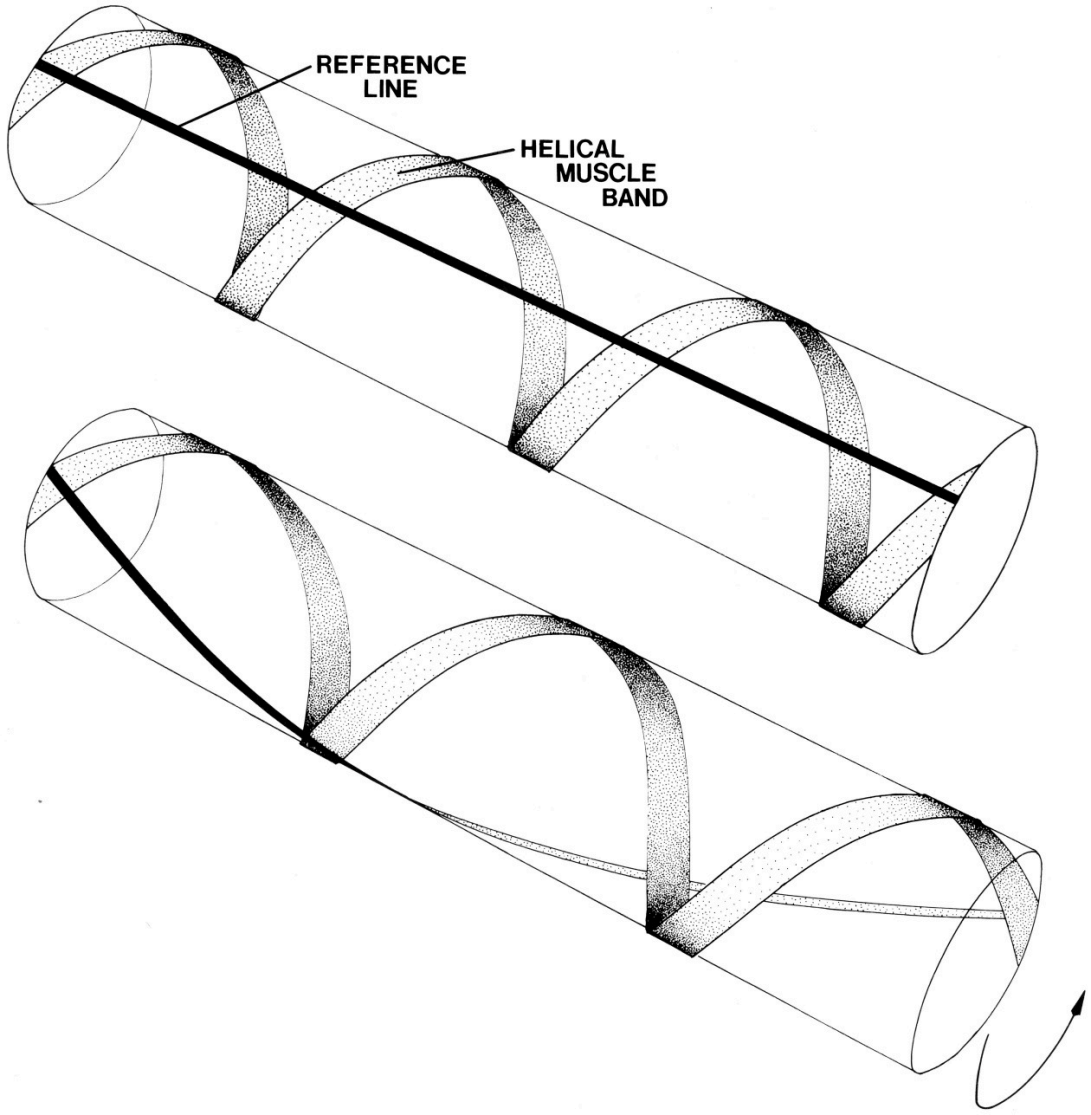
**Schematic diagram illustrating the effect of contraction of helical muscle**. A single left-hand helical muscle band is illustrated with a longitudinal black line for reference. Upon shortening of the muscle the cylinder twists. The direction of torsion depends on the handedness of the helical muscle band. From Kier (1982).

The right- and left-handed helical muscle layers are responsible for torsion or twisting of the tentacles around their longitudinal axis (Figure 3; Kier, 1982). During the elongation phase of the tentacle strike, the tentacles often twist and are observed to be capable of twisting in either direction, depending on prey orientation. This torsion appears to be important in orienting the tentacle club so that the side equipped with suckers strikes the prey. A biomechanical analysis shows that the helical muscle layers cause torsion; the direction of torsion depends on the handedness of the helical muscle layer contracting (Kier, 1982). The helical muscle layers must accommodate changes in the helical path length as the tentacle elongates and shortens. As the tentacle extends from the fully contracted state, the helical muscles must themselves shorten until their fiber angle reaches 54°44′. As the tentacles elongate beyond this point and the fiber angle decreases further, the helical muscles are elongated. The peripheral location of the helical muscle layers provides a larger moment through which torque can be applied than more central location (Kier and Smith, 1985).

### Squid and cuttlefish arms

The arms of squid and cuttlefish serve important roles in prey handling, manipulation of objects, swimming, and reproduction. Unlike the specialized prey capture tentacles, the arms do not undergo significant length change. Instead, many of the tasks performed require bending movements, both with generalized bends over the entire length of the arm and also more localized bending movements. Torsion or twisting around the longitudinal axis is also common (Kier, 1982).

#### Morphology and microanatomy of the musculature of squid and cuttlefish arms

**Figure 4:**
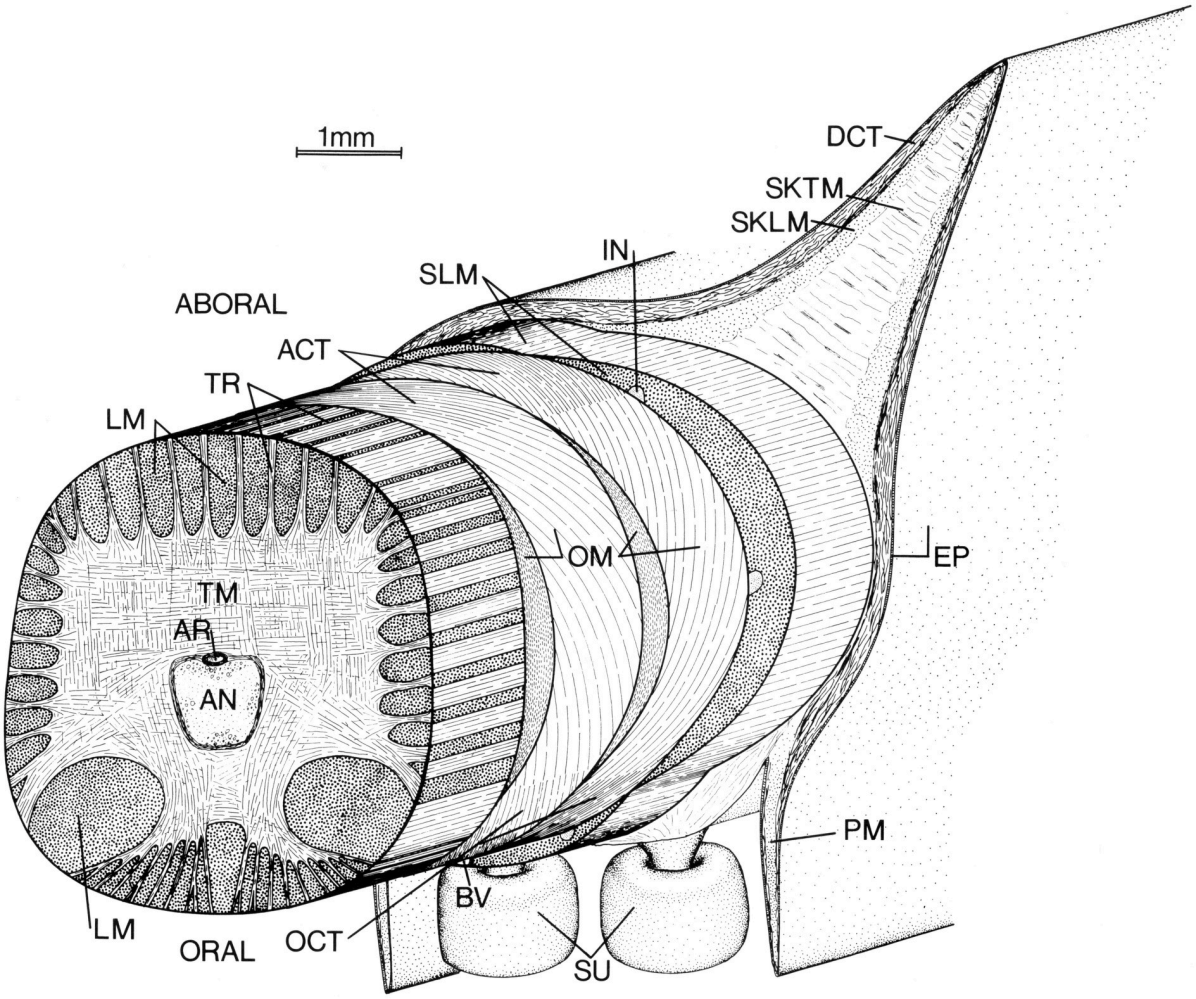
**Schematic diagram of left arm of a loliginid squid**. AN, axial nerve cord; ACT, aboral connective tissue (fibrous); AR, artery; BV, superficial brachial vein; DCT, dermal connective tissue; EP, epithelium; IN, intramuscular nerve cord; LM, longitudinal muscle; OCT, oral connective tissue (fibrous); OM, oblique muscle; PM, protective membrane; SKLM, swimming keel longitudinal muscle; SKTM, swimming keel transverse muscle; SLM, superficial longitudinal muscle; SU suckers; TM, transverse muscle; TR, trabeculae of transverse muscle. From Kier (1982).

As in the tentacles described above, the axial nerve cord that runs longitudinally down the central axis of the arms is surrounded by a mass of transverse muscle (Figure 4; Guérin, 1908; Kier, 1982). Muscle fibers in the transverse muscle mass are arranged in planes perpendicular to the longitudinal axis of the arm. Bundles of these fibers extend between bundles of longitudinal muscle as sheets of fibers called trabeculae by Graziadei (1965). After passing between the longitudinal muscle bundles orally and aborally (the surface of the arm with suckers is termed “oral”) they insert on fibrous connective tissue sheets. The muscle fiber bundles that extend laterally insert on the connective tissue surrounding the oblique muscles of the arm (Kier, 1982).

The oblique muscles located on each side of the arm have their origin and insertion on the oral and aboral fibrous connective tissue sheets. The fibers in the connective tissue sheets are arranged in a crossed fiber array with half of the fibers arranged as a right-hand helix and the other half of the fibers arranged as a left-hand helix. The fibers are oriented at a fiber angle of 72° with the longitudinal axis of the arms. The muscle fibers of the oblique muscle pair are oriented with the same fiber angle as the connective tissue fibers to which they are connected. The oblique muscles and associated connective tissue layers thus form a composite right- and left-handed helix of muscle fibers and connective tissue fibers (Kier, 1982).

Surrounding the oblique muscles and associated connective tissue sheets are three bundles of longitudinal muscle, one located orally and the others laterally. The aboral surface of the arms also includes longitudinal fin-like projections called swimming keels. The cores of the swimming keels consist of non-fibrous connective tissue with scattered muscle bundles that extend transversely across the keel and longitudinal muscle fibers that extend as a sheet over the core. Projecting from the oral surface of the arm are the rows of suckers, which are enclosed on each side by protective membranes. (Girod, 1884; Niemiec, 1885; Nixon and Dilly, 1977; Kier, 1982).

The arm is covered by a loose connective tissue dermis that contains chromatophores, iridophores, blood vessels and nerves. A simple cuboidal-to-columnar epithelium covers the dermis.

#### Biomechanics of support and movement in squid and cuttlefish arms

**Figure 5:**
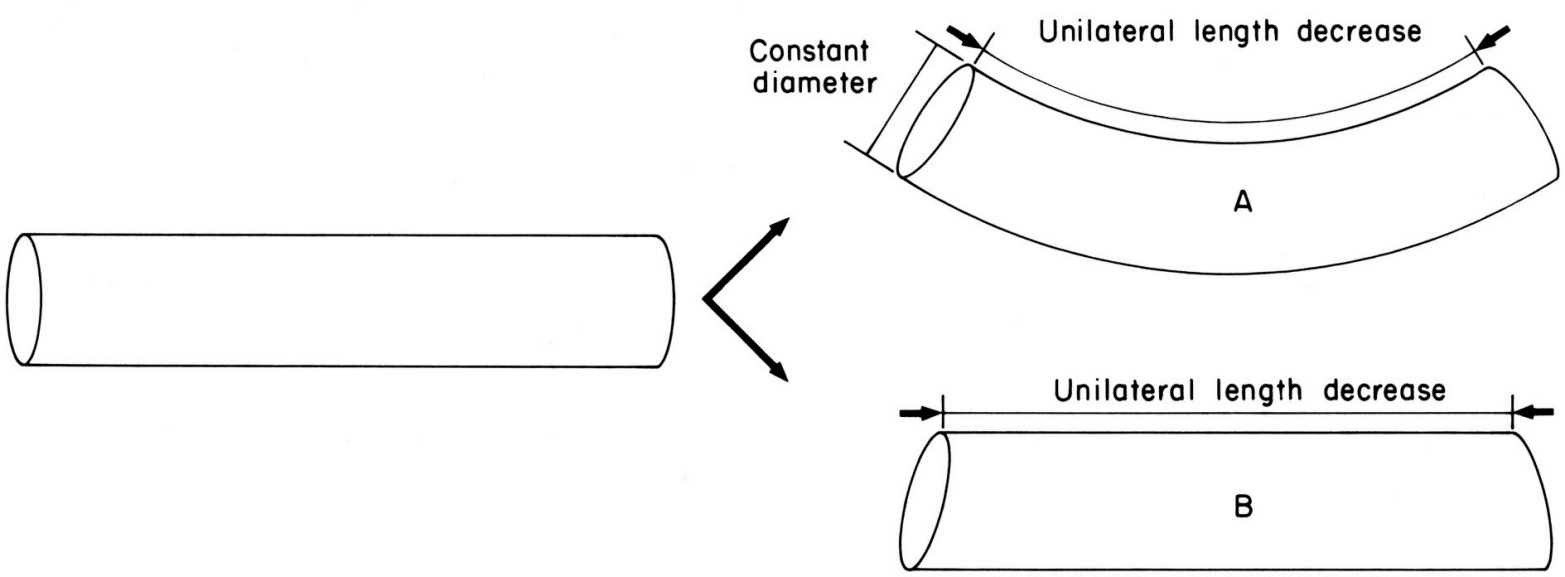
**Diagram illustrating the requirements for active bending**. Unilateral length decrease is caused by contraction of longitudinal muscle on one side. In case A, constant diameter is maintained thereby providing resistance to longitudinal compression and causing bending. Constant diameter can be maintained by contractile activity of the transverse muscle. In case B, constant diameter is not maintained and without resistance to longitudinal compression the structure is shortened but not bent. From Kier and Smith (1985).

**Figure 6:**
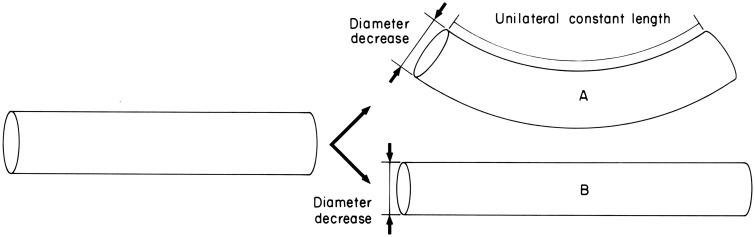
**Diagram illustrating the requirements for active bending**. Diameter decrease is caused by contraction of the transverse muscle. In case A, unilateral constant length is maintained by contractile activity of the longitudinal muscle on one side and thereby causes bending. In case B, unilateral constant length is not maintained and diameter decrease simply causes elongation. From Kier and Smith (1985).

One of the most important arm movements, bending, requires selective contraction of the longitudinal muscle on the side of the arm representing the inside radius of the bend. Since longitudinal muscle bundles are present around the entire periphery of the cross-section, bending in any plane is possible, although especially large longitudinal muscle bundles are present on the oral side of the cross section and forceful bending in the oral direction is particularly important in prey handling. Longitudinal muscle contraction creates a longitudinal compressional force that would tend to simply shorten the arm, rather than bend it, without some mechanism to resist this force (Kier, 1982; Kier and Smith, 1985). The resistance to volume change of the tissue of the arm is crucial for providing this resistance to longitudinal compression. Because the arm is essentially constant in volume, any shortening would result in an increase in the diameter; to resist the longitudinal compressional force an increase in diameter must be prevented (Figure 5). The transverse muscle is oriented so that it can control the diameter of the arm and thus can provide the resistance to longitudinal compression that is required for bending. Active arm bending therefore requires simultaneous contractile activity in both the longitudinal and the transverse muscle fibers of the arm. In the situation described above, the transverse muscle maintains the diameter, resisting longitudinal compression while the longitudinal fibers shorten one side of the arm. Bending can also be caused by decreasing the diameter due to shortening of the transverse muscle, as long as the longitudinal muscle on one side of the arm (again, the inside radius of the bend) resists elongation (Figure 6). The relative contribution of shortening of the transverse or longitudinal muscle to bending probably varies and the two situations described above represent endpoints on a continuum. The longitudinal muscle bundles are situated peripherally in the arm, which increases the bending moment compared with a more central location close to the neutral plane (the neutral plane of a bending beam is where all bending stresses are zero and is usually in the center; Kier and Smith, 1985).

The torsional force that is required to twist the arms is provided by the oblique muscles and the associated crossed-fiber connective tissue sheets (Kier, 1982). Both right- and left-handed muscle and connective tissue fiber layers are present. The fibers of a given handedness can be considered as a composite of connective tissue fibers alternating with muscle fibers that wrap the arm helically along the length. Contraction of one of the composite systems will twist the arm, with the direction of twist depending on the handedness of the helical fiber system. The torsional stiffness of the arm can be increased with contractile activity of both the right- and left-handed oblique muscle systems. Active control of torsional stiffness is particularly important while handling struggling prey. The placement of the oblique muscle in a peripheral location provides a larger moment through which the torque can be applied than a more central location close to the neutral axis (the neutral axis is located at the center of a beam in torsion and does not experience shear stress) (Kier and Smith, 1985).

### Octopodid arms

The eight arms of octopuses serve a variety of functions including prey capture, locomotion, manipulation of objects, grooming, burying, copulation, defense, chemosensing, and tactile sensing. They incorporate all of the movements exhibited by both the arms and the tentacles of decapod cephalopods; they undergo significant elongation and shortening, are capable of complex and diverse bending and curling movements, and also create torsional movements in either direction. The arms are, in addition, capable of active control of stiffness. Indeed, their capabilities have attracted the attention of robotics engineers as inspiration for the design and construction of a new class of robotic arms (McMahan et al., 2004, 2006; Walker et al., 2005; Jones and Walker, 2006a,b; Calisti et al., 2011; Kang et al., 2012; Laschi et al., 2012; Cianchetti et al., 2015).

#### Morphology and microanatomy of the musculature of octopodid arms

Three divisions of the musculature of the arms of octopuses were recognized by Graziadei (1965, 1971) including (1) the intrinsic musculature of the suckers (Kier and Smith, 1990, 2002; Tramacere et al., 2013); (2) the intrinsic musculature of the arms, and (3) the acetabulo-brachial musculature connecting the suckers to the arm musculature. The focus of this section will be on the intrinsic musculature of the arms (Colasanti, 1876; Guérin, 1908; Tittel, 1961, 1964; Socastro, 1969; Kier and Stella, 2007; Feinstein et al., 2011) based primarily on observations of *Octopus bimaculoides, Octopus briareus*, and *Octopus digueti* (Kier and Stella, 2007).

**Figure 7:**
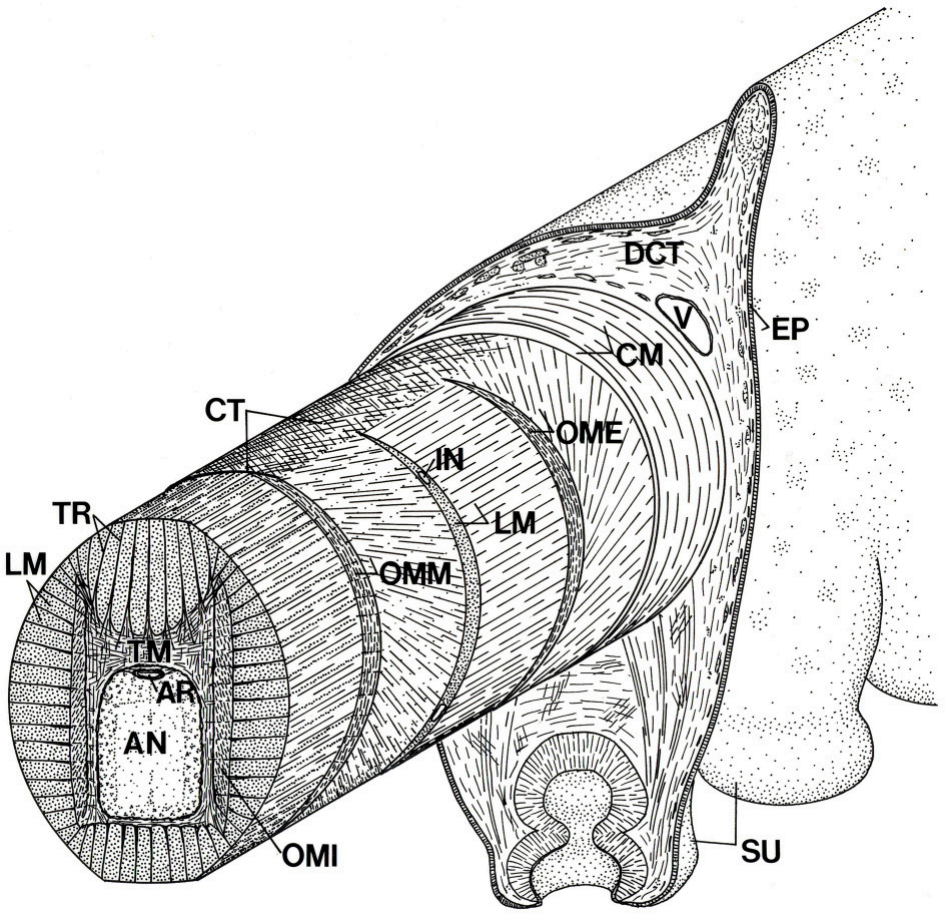
**Schematic diagram of the arm of *Octopus* showing the three-dimensional arrangement of muscle fibers and connective tissue fibers**. AN, axial nerve cord; AR, artery; CM, circumferential muscle layer; CT, connective tissue; DCT, dermal connective tissue; EP, epidermis; IN, intramuscular nerve; LM, longitudinal muscle fibers; OME, external oblique muscle layer; OMI, internal oblique muscle layer; OMM, median oblique muscle layer; SU, sucker; TM, transverse muscle fibers; TR; trabeculae; V, vein. From Kier (1988).

As in the arms and tentacles of decapod cephalopods described above, the axial nerve cord extends longitudinally down the arm and is surrounded by the muscle fibers of the transverse muscle mass, with fibers oriented in planes perpendicular to the longitudinal axis of the arm (Figure 7). Bundles of muscle fibers of the transverse muscle mass are arranged approximately orthogonally, either extending from the oral to aboral surface or at right angles to this and thus from side to side. The transverse muscle fiber bundles that extend from the oral to the aboral surface originate on thick crossed-fiber connective tissue sheets on the oral and aboral sides of the arm. The fiber bundles project toward the central axis of the arm in longitudinal sheets termed trabeculae that extend between bundles of longitudinal muscle fibers. Many insert on a fibrous connective tissue layer surrounding the axial nerve cord or extend to insert on the fibrous connective tissue sheet on the opposite side of the arm. The transverse muscle fiber bundles that extend from side to side in the arm originate on connective tissue surrounding the external oblique muscles located on each side on the arm and pass through the longitudinal and oblique muscles in the form of trabeculae between the longitudinal muscle bundles or as individual bundles through the oblique muscle. Many insert on the connective tissue surrounding the axial nerve cord. Some of the transverse muscle fiber bundles that run from side to side pass oral, and especially aboral, to the axial nerve cord and extend to the opposite side to insert on the connective tissue surrounding the external oblique muscle (Kier and Stella, 2007). The orientation of transverse muscle fibers perpendicular to the long axis of the arm may not, however, be universal for octopuses; Feinstein et al. (2011) report that transverse muscle fibers in the arm of *Octopus vulgaris* are not restricted to the transverse plane of the arm.

Longitudinal muscle fiber bundles extend the length of the arm between the trabeculae of the transverse muscle. The bundles are present on all sides of the transverse muscle mass so the entire periphery of the cross-section includes longitudinal muscle bundles, although the cross-sectional area of the aboral quadrant is larger than the other quadrants. A crescentic shaped layer of longitudinal muscle is present between the median and external oblique muscles (Kier and Stella, 2007).

Three sets of oblique muscle fibers are present on each side of the arm. The external oblique muscles enclose the intrinsic muscle of the arm and are the most superficial. The median oblique muscles are more central and are separated from the external oblique muscles by longitudinal muscle fibers as described above. The internal oblique muscles are the most central and are located on each side of the core of transverse muscle. The handedness of a given oblique muscle is opposite to that of the other member of the pair on the opposite side of the arm. In addition, on a given side, the handedness of the external and internal oblique muscles is the same and is opposite to that of the median oblique muscle. The external and the median oblique muscles have their origin and insertion on the oral and aboral connective tissue sheets. The fiber angles of the median and external oblique muscles are similar to the fibrous connective tissue layers to which they attach (mean angles for *O. bimaculoides* of 63–74°; Kier and Stella, 2007). The fibers of the inner oblique muscles do not show a distinct origin and insertion and instead appear to interdigitate with the longitudinal and transverse musculature. The fiber angle of the inner oblique muscles is lower than that of the median and external oblique muscles [mean angles range from 42° to 56° in species measured (Kier and Stella, 2007).

Surrounding the intrinsic muscle of the arm is a thin layer of circular muscle with fibers arranged circumferentially around the arm. The layer is thickest on the aboral side of the arm, covers the aboral connective tissue sheet and extends toward the oral side of the arm, wrapping the external oblique muscles and inserting on the oral connective tissue sheet (Kier and Stella, 2007).

#### Biomechanics of support and movement in octopus arms

Support and movement in octopus arms is achieved in a similar manner to that described above for the arms and tentacles of decapods and relies on the resistance to volume change of the musculature of the arms. The arms are capable of a remarkable diversity and complexity of movements (Gutfreund et al., 1996, 1998; Mather, 1998; Huffard et al., 2005; Sumbre et al., 2005, 2006; Yekutieli et al., 2005a,b; Levy et al., 2015), all of which are produced by some combination of the four basic arm deformations described earlier: elongation, shortening, bending, and torsion. Octopus arms are notable because these deformations may be quite localized or they may occur over the entire length of the arm. In addition, they may occur at one location or at multiple locations on an individual arm. Bending movements can occur in any plane and torsional movements are observed in either direction. The stiffness in tension, compression, bending and torsion is also under active control by the animal (Kier and Stella, 2007).

Since the arm tissue resists volume change, a decrease in cross section must result in an increase in length. This decrease in cross-section is likely created by contraction of the muscle fibers of the transverse muscle mass. The elongation created can either be localized, involving only a portion of the transverse muscle, or it can occur over the entire length of the arm. The thin circular muscle layer is also oriented so that its contraction will elongate the arm, but its physiological cross-sectional area is quite small and thus the force it could produce for elongation is small. One possible role for the circular muscle layer is in providing arm tonus for maintaining posture (Kier and Stella, 2007).

Shortening likely involves contraction of the longitudinal muscle bundles that extend the entire length of the arm. Since the arm resists volume change, shortening of the arm results in an increase in cross-section and thus causes elongation of the transverse and circular muscle fibers. The transverse and longitudinal muscle fibers thus function as antagonists and produce the force required for re-elongation of one another (Kier and Stella, 2007).

The muscle activation required for bending movements is similar to that described above for bending of decapod arms. Active bending requires selective contraction of the longitudinal muscle bundles along the side of the arm that represents the inside radius of the bend. The support required to resist the longitudinal compressional force that would otherwise simply shorten the arm is provided by the transverse muscle mass. Active bending movements thus require simultaneous contraction of the transverse and longitudinal muscle. Bending may also occur if the transverse muscle decreases the cross-section while the longitudinal muscle on one side of the arm (again, the inside radius of the bend) maintains a constant length. As described above for decapod arms, the two examples provided here probably represent endpoints on a continuum of relative shortening of the transverse and longitudinal muscle. Abrupt bends, as have been observed in some behaviors (Sumbre et al., 2005, 2006), probably involve selective localized contraction of the longitudinal and transverse arm musculature while more gentle bends likely involve more widely distributed muscle activity.

The arms of octopus provide an interesting contrast to both the tentacles and arms of decapods. As described above, the tentacles function primarily in elongation and shortening while the arms of decapods exhibit little length change and instead produce bending movements. The arms of octopuses incorporate both bending and length change (Hanassy et al., 2015). This can be achieved using the same musculature, the transverse and longitudinal muscle fibers, by simply altering their pattern of activity; sequential activity during elongation and shortening and simultaneous activity during bending (Kier and Stella, 2007).

Based on simple engineering considerations, the force generated by the arm during bending movements is greater if the longitudinal muscle fibers are located as far as possible from the neutral plane of the arm. The longitudinal muscle is indeed located away from the central axis of the arm. In addition, longitudinal muscle bundles are located around the entire periphery of the cross-section of the intrinsic muscle which allows bending stresses to be exerted in any plane. The transverse muscle is most robust in the aboral portion of the arm, which is consistent with its role in supporting and producing oral bending (the most common mode of forceful bending) in conjunction with the longitudinal muscle bundles on the oral side (Kier and Smith, 1985; Kier and Stella, 2007).

In addition of active bending movements, co-contraction of the transverse and longitudinal muscle increases the flexural stiffness of the arm. Such a pattern of activation is a component of the reaching behavior that has been described by Hochner, Flash and coworkers (Gutfreund et al., 1996, 1998; Yekutieli et al., 2005a,b). In some arm movements, the arm is stiffened and then rotated at its base by the musculature of the web at the base of the arms (Guérin, 1908).

As in the arms of decapods, torsional movements are generated by contraction of the oblique muscles. The crossed fiber helical connective tissue arrays are a key component of the helical system of muscle and connective tissue as they transmit the force generated by the oblique muscles. The external and median oblique muscle pairs on each side of the arm and the associated cross-fiber connective tissue array represent both a left- and a right-handed helical system and thereby allow torsional forces to be generated in either direction, consistent with observations of twisting of the arms in either direction. Co-contraction of the external and median oblique muscle systems likely increases the torsional stiffness of the arm. The torsional moment is greater if the oblique muscles are located as far from the neutral axis as possible. The external and median oblique muscles are indeed located away from the neutral axis. The functional role of the internal oblique muscles is unclear since they are more central so would be less effective in generating a torsional moment and they have the same handedness as the external oblique. Future work involving electromyography of the internal oblique muscles during arm movement and force production would be of interest in order to determine their biomechanical role (Kier and Stella, 2007).

Octopus arms provide an example of the highly localized movements and deformations that are possible in appendages that rely on muscular hydrostatic mechanisms. In comparison with a conventional hydrostatic skeleton, localized activation of muscle fibers has a localized effect, rather than the more generalized effect of increasing the hydrostatic pressure of a large fluid filled cavity. Deformations can occur in many directions at any location or at multiple locations and the arms must therefore have the neuromuscular control required to activate selectively small groups of muscle fibers and to precisely modulate their force production. Indeed, the motor units of the transverse and longitudinal muscle are small and there does not appear to be electrical coupling between the fibers (Matzner et al., 2000). In addition, muscle fiber activation can be controlled directly by neural activity, thereby providing precise modulation of muscle force production (Matzner et al., 2000; Rokni and Hochner, 2002). The difficulty with such a system, however, is the potential complexity of motor control that is required. Recent studies are providing important insights into motor pathways and mechanosensory mechanisms (Gutfreund et al., 2006) and mechanisms that may simplify the neuromuscular control of the arm (Gutfreund et al., 1996; Sumbre et al., 2001, 2005, 2006; Zullo and Hochner, 2011; Hochner, 2012, 2013; Levy et al., 2015).

## Ultrastructure and specialization of the muscle of cephalopod arms and tentacles

### Ultrastructure of cephalopod muscle

**Figure 8:**
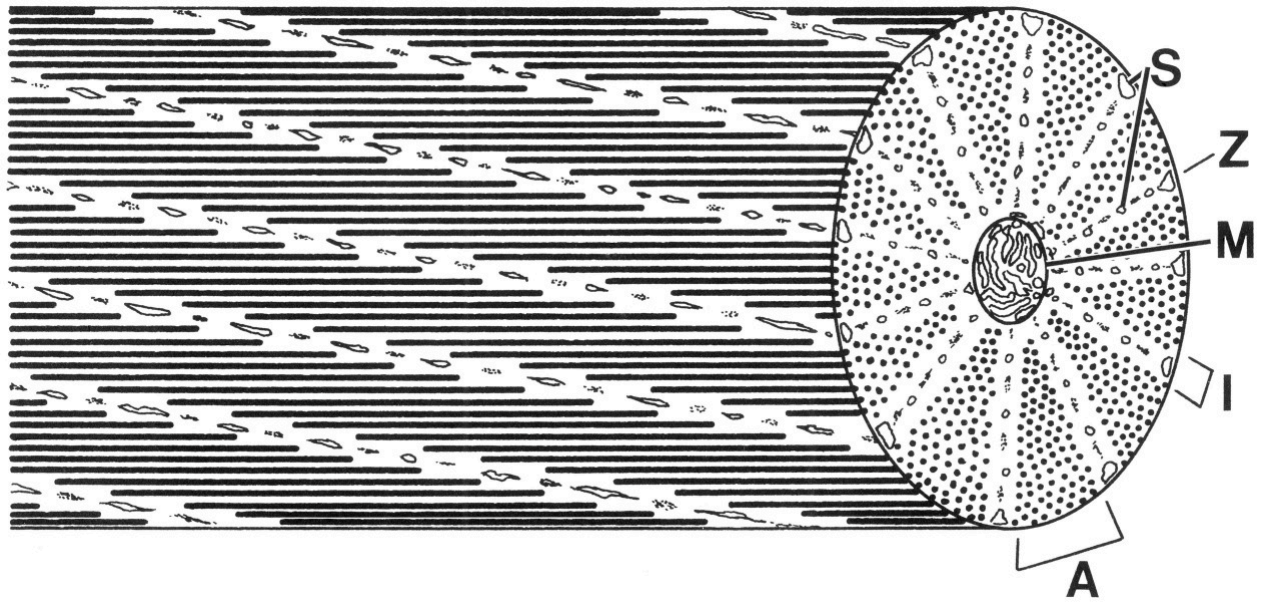
**Schematic diagram of a cephalopod obliquely striated muscle fiber**. Note that a cross-section of an obliquely striated muscle cell shows an analogous sequence of bands to those seen in a longitudinal section of a cross-striated fiber. A, A-band; I, I-band; M, mitochondria; S, sarcoplasmic reticulum; Z, elements. From Kier (1996).

The majority of the musculature of the arms and tentacles of coleoid cephalopods, and indeed that of the entire animal, is obliquely striated (Hanson and Lowy, 1960; Hoyle, 1964, 1983; Amsellem and Nicaise, 1980; Chantler, 1983; Nicaise and Amsellem, 1983; Budelmann et al., 1997). This striation pattern is common in the invertebrates, occurring in at least 14 phyla (Thompson et al., 2014) and differs from the more familiar cross-striated muscle fibers of the vertebrates and arthropods because the myofilaments, although parallel to the long axis, are not lined up in register across the fiber (Figure 8). In obliquely striated muscle fibers, the thick and thin myofilaments are arranged in a staggered array, forming a helical or oblique pattern of A-bands (containing thick filaments), I-bands (containing thin filaments) and Z material or dense bodies (anchor the thin myofilaments). Thus, the fibers lack the transverse banding pattern observed in longitudinal section that characterizes cross-striated muscle fibers. In transverse sections, however, the fibers show a similar sequence of banding to that observed in longitudinal sections of cross-striated muscle; the staggered arrangement of myofilaments means that a single transverse section passes through I-bands, A-bands, and Z material in a single fiber. The myofilaments surround a central core containing mitochondria and the single cell nucleus. The size of the mitochondrial core varies. In the mantle and in the fins of squids there are distinct zones that include either mitochondria-rich fibers with large cores or fibers with fewer mitochondria and smaller cores. The mitochondria rich fibers are analogs of the red muscle of vertebrates, operating primarily aerobically and used in repetitive movements, while the mitochondria-poor fibers are anaerobic, white muscle analogs that are recruited for short term maximal efforts (Bone et al., 1981; Mommsen et al., 1981; Kier, 1989; Kier et al., 1989; Johnsen and Kier, 1993; Bartol, 2001). The fibers of the arms and tentacles of coleoids that have been studied are predominantly the mitochondria-poor fibers, but additional work is needed to examine this issue. The striation angle, defined as the angle between the longitudinal axis and the alignment of Z elements, is quite low, ranging from 6 to 12° at rest (Hanson and Lowy, 1957). The striation angle increases as the fiber shortens and decreases as the fiber is elongated.

It is likely that in bilaterians, striated muscle evolved independently multiple times (Oota and Saitou, 1999; Schmidt-Rhaesa, 2007; Burton, 2008; Chiodin et al., 2011; Steinmetz et al., 2012). There are examples both of derivation of cross-striation from oblique striation and derivation of oblique striation from cross-striation (Schmidt-Rhaesa, 2007). Thus, ultrastructural similarity does not necessarily indicate common evolutionary origin and the evolutionary relationships of eumetazoan striated muscle remain unclear (Steinmetz et al., 2012).

### Ultrastructure of the transverse muscle mass of the arms of decapods and octopodids

The fibers of the transverse muscle mass of the arms of the loliginid squid *Doryteuthis pealeii* and the ommastrephid squid *Illex illecebrosus* are obliquely striated (Kier, 1985). These fibers have been examined in the most detail so the description that follows will focus on their ultrastructure. Recent preliminary investigations of the ultrastructure of the fibers of the transverse muscle mass of the arms of the cuttlefish *Sepia officinalis* and of *Octopus bimaculoides* (Shaffer and Kier, 2016) have shown similar ultrastructure to that of the arms of squid but additional work is needed.

**Figure 9:**
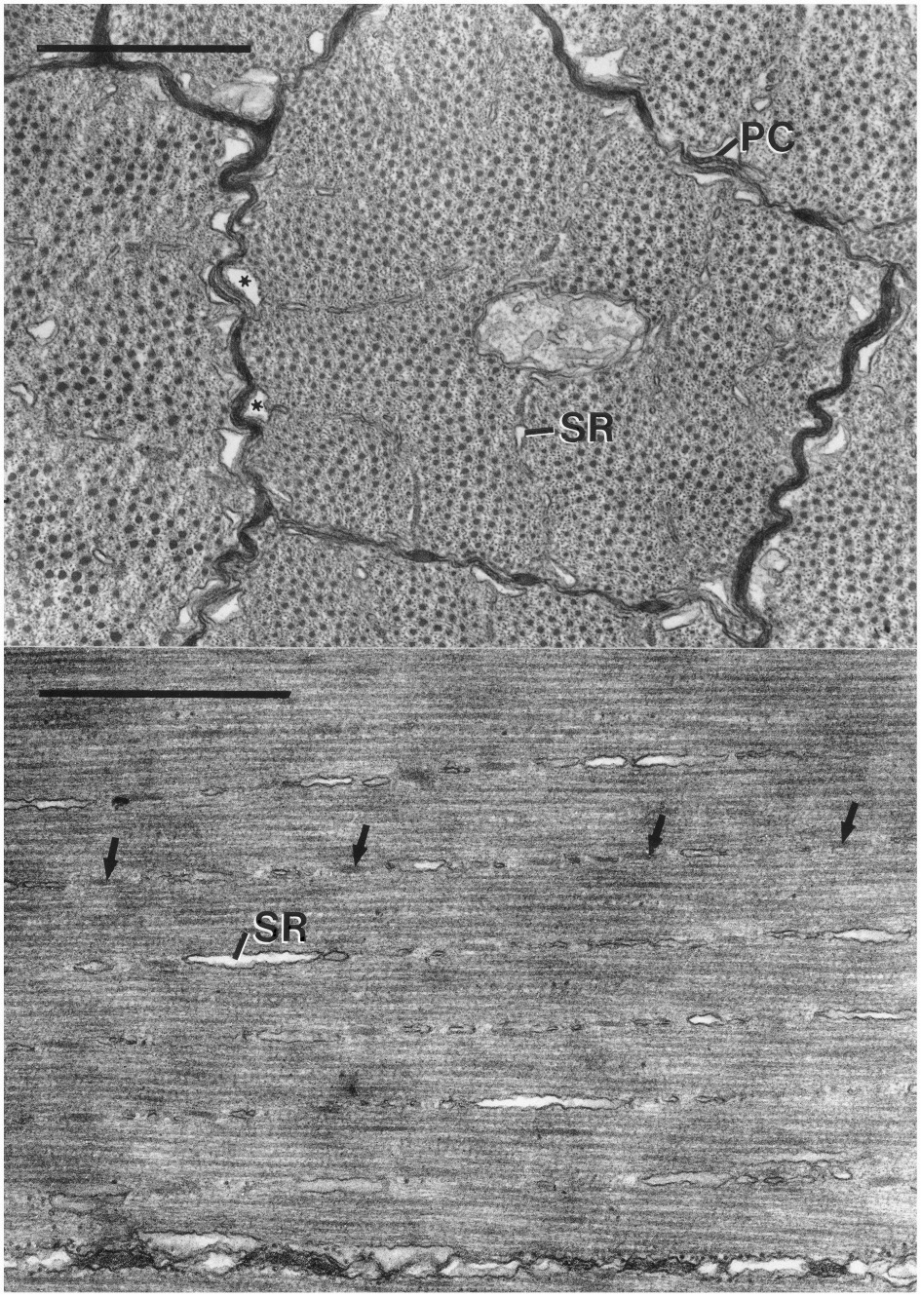
**Top: Electron micrograph of transverse section of obliquely striated muscle fibers of the transverse muscle of the arm of *Doryteuthis pealeii***. The outer membranes of the terminal cisternae (^*^) make specialized contacts or peripheral couplings (PC) with the sarcolemma. Regularly spaced junctional feet are visible in the peripheral coupling labeled PC. The terminal cisternae occur where the Z elements and associated sarcoplasmic reticulum (SR) approach the sarcolemma. The scale bar length equals 1 μm. **Bottom:** Electron micrograph of longitudinal section of obliquely striated muscle fibers of the transverse musculature of the arm of *Illex illecebrosus*. The long axis of the muscle fiber is oriented horizontally on the page. The intramyoplasmic zones of sarcoplasmic reticulum (SR) and dense bodies (arrows) are oriented at a small angle with respect to the horizontally oriented thick filaments. The scale bar length equals 1 μm. From Kier (1985).

The fibers are short (typically less than a millimeter), are circular or polygonal in cross-section and show a range of diameters (mean = 3.4 μm, *SD* = 1.1 μm for *I. illecebrosus* and mean = 2.8 μm, *SD* = 0.9 μm for *D. pealeii*) in part because the cells are fusiform in shape and a given section plane cuts the fibers at various points along their length (Figure 9). The fibers are surrounded by an amorphous, electron-dense extracellular material (Kier, 1985; Feinstein et al., 2011).

The sarcoplasmic reticulum is present in three zones. A peripheral zone of sarcoplasmic reticulum is present in the sarcoplasm adjacent to the sarcolemma. Specialized peripheral couplings are present between the sarcolemma and the outer portion of the membrane of the terminal cisternae of the sarcoplasmic reticulum in this zone and are common where the Z elements are adjacent to the sarcolemma. Regularly spaced junctional feet are present in the space between the sarcolemma and the membrane of the sarcoplasmic reticulum. A second zone of sarcoplasmic reticulum is present in the plane of the Z elements and consists of a network of units that are elongated parallel to the longitudinal axis of the fiber. This intramyoplasmic zone of sarcoplasmic reticulum is interspersed between the dense bodies that form the Z material in these cells. A third zone of sarcoplasmic reticulum is present surrounding the mitochondrial core. The fibers lack a transverse tubular system so the peripheral couplings described above likely function in excitation contraction coupling in a manner similar to that of the triad of a vertebrate skeletal muscle fibers (Kier, 1985).

The diameter of the thick filaments is greatest at their midpoint (mean = 31.1 nm, *SD* = 1.9 nm for *I. illecebrosus* and 36.0 nm, *SD* = 3.0 nm for *D. pealeii*) and decreases as they taper at each end. It is challenging to obtain accurate measurements of thick filament length in these cells due to the difficulty of obtaining exactly longitudinal sections. In a study where special care was taken during sectioning, the thick filaments of the transverse muscle mass of the arms of *D. pealeii* were measured to be 7.4 μm long (*SD* = 0.44 μm; Kier and Curtin, 2002).

### Ultrastructure of the transverse muscle mass of the tentacles of decapods

The muscle fibers of the transverse muscle mass of the tentacles of *D. pealeii* and *I. illecebrosus* are unusual for cephalopods because they show cross-striation (Kier, 1985, 1991). Recent preliminary investigation of the transverse muscle mass of the tentacles of the cuttlefish *S. officinalis* shows similar ultrastructure with cross-striations (Shaffer and Kier, 2016).

**Figure 10:**
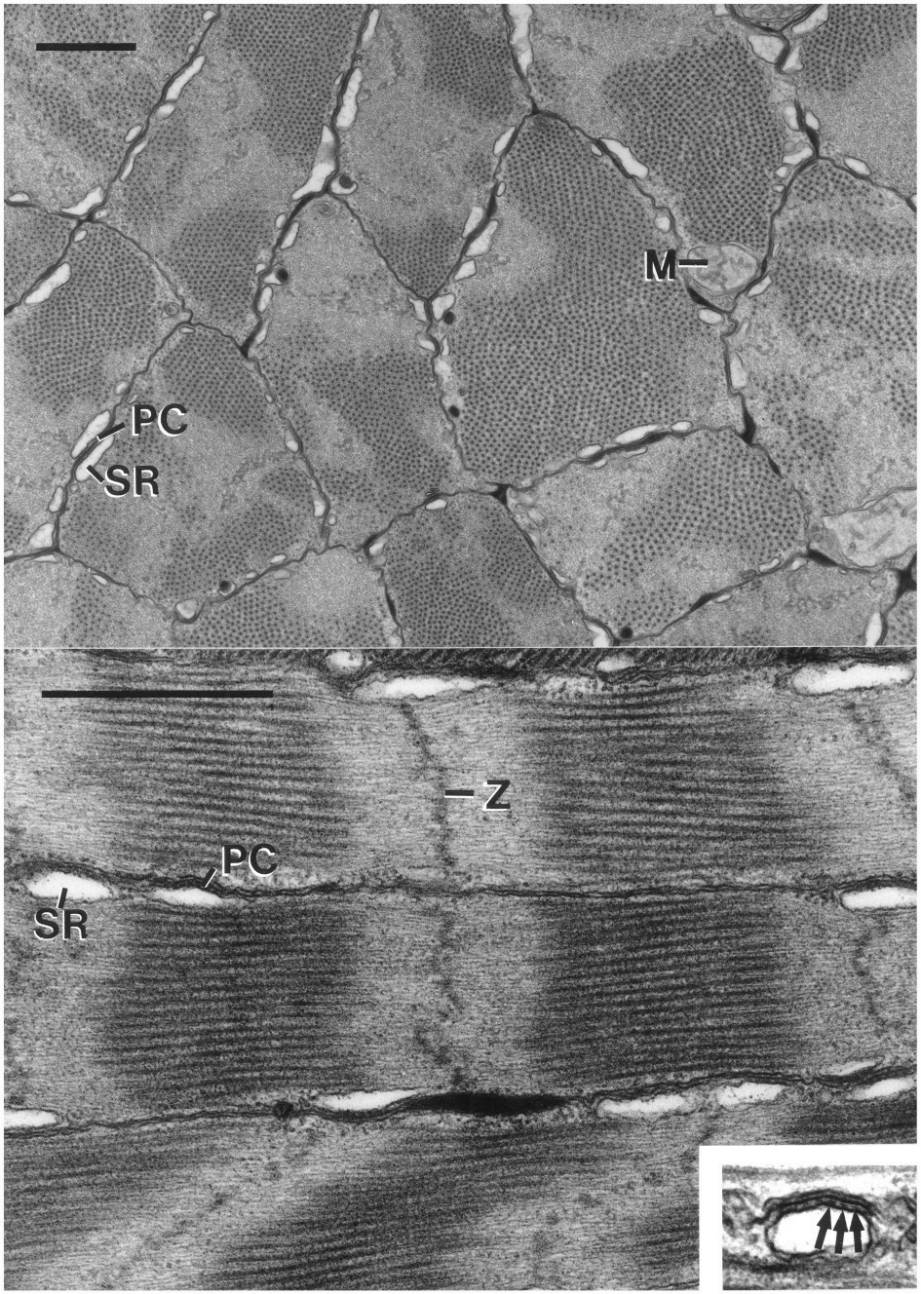
**Top: Electron micrograph of transverse section of the cross-striated muscle fibers of the transverse muscle mass of the tentacle of *Doryteuthis pealeii***. Mitochondria (M) are located immediately beneath the sarcolemma. The outer membrane of the sarcoplasmic reticulum (SR) makes specialized contacts or peripheral couplings (PC) with the sarcolemma. Note that the A band (thick filaments in cross-section) passes in and out of the section plane in a single fiber. The scale bar length equals 1 μm. **Bottom:** Electron micrograph of longitudinal section of cross-striated muscle fibers of the transverse musculature of the tentacle of *Doryteuthis pealeii*. The outer membrane of the sarcoplasmic reticulum (SR) forms peripheral couplings (PC) with the sarcolemma. The inset shows a higher magnification view of a peripheral coupling in which junctional feet (arrows) are visible. Note that the Z-disc (Z) is diffuse and sometime follows an angled course across the fiber. The scale bar length equals 1 μm and the inset is 0.5 μm wide. From Kier (1985).

The fibers are short (typically less than a millimeter), small diameter cells (mean diameter = 2.1 μm, *SD* = 0.5 μm for *I. illecebrosus* and 2.4 μm, *SD* = 0.7 μm for *D. pealeii*; Figure 10). Unlike the obliquely striated cells of the arms, the mitochondria are not in the core and instead are located peripherally in the cell, immediately beneath the sarcolemma. The tubules of the sarcoplasmic reticulum are restricted to the same area as the mitochondria, immediately beneath the sarcolemma. The cells thus lack transverse tubules (invaginated tubules) and the fibers are not subdivided into myofibrils. The sarcoplasmic reticulum forms specialized couplings with the sarcolemma in a manner similar to that described above for the obliquely striated fibers of the arms. The coupling includes regularly spaced electron dense junctional feet in the space between the outer membrane of the sarcoplasmic reticulum and the sarcolemma. The peripheral couplings of one fiber are often aligned with those of adjacent fibers (Kier, 1985).

The diameter of the thick filaments at their midpoint is smaller than that of the obliquely striated fibers (mean = 23.7 nm, *SD* = 1.0 nm for *I. illecebrosus* and 31.4 nm, *SD* = 2.2 nm for *D. pealeii*) and they do not appear to taper at their ends. The thick filaments have an electron-lucent core when observed in transverse section, in contrast to the core of the thick filaments in the obliquely striated muscle fibers of the arms, which are electron dense. This may be related to the greater paramyosin content of the thick filaments of the obliquely striated muscle fibers since paramyosin occupies the core (Kier and Schachat, 1992). The thick filament length of the transverse muscle fibers of *D. pealeii* was measured to be only 0.81 μm (*SD* = 0.08 μm) (Kier and Curtin, 2002). The fibers lack an M band, a structure present in vertebrate and arthropod cross striated muscle which is located in the center of the A band where thick filaments are bound together by cross-links. The sarcomeres of the tentacle fibers are often observed to be sheared so that the Z disc, A bands, and I bands are not perpendicular to the long axis and instead follow an angled or curved course across the diameter (Kier, 1985).

The Z disc of the transverse muscle fibers of the tentacles is not as regularly arranged as it is in vertebrate and arthropod muscle fibers and instead appears to be a loose grouping of electron-dense material rather than the organized network observed in the Z discs of vertebrates and arthropods (Kier, 1985).

### Development and differentiation of the transverse muscle of the arms and tentacles

**Figure 11:**
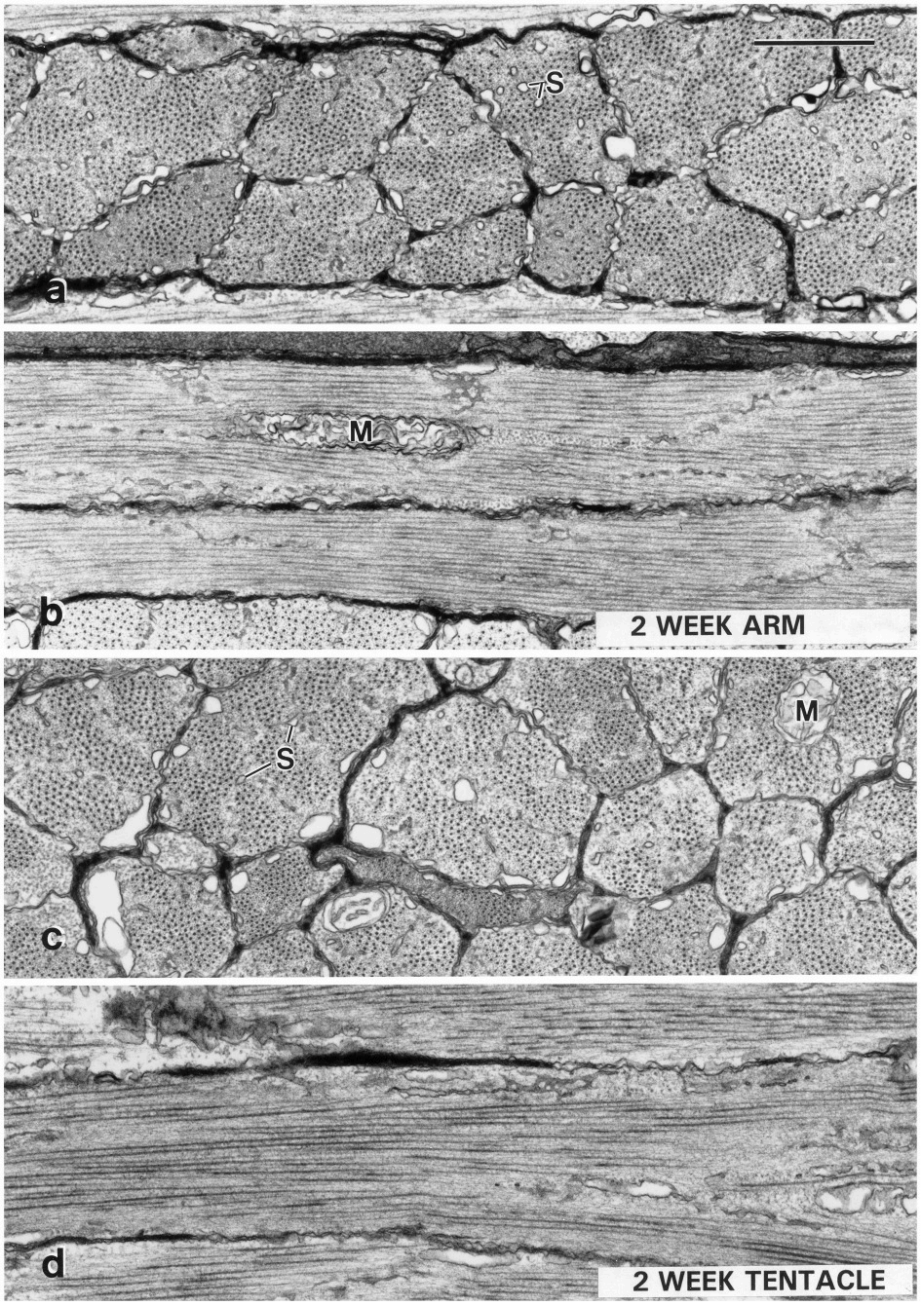
**Transmission electron micrographs of muscle cells from a 2-week-old *Sepioteuthis lessoniana* (dorsal mantle length = 11 mm)**. Transverse **(A)** and longitudinal **(B)** sections of fibers from the transverse muscle of the arm (arm III), and transverse **(C)**, and longitudinal **(D)** sections of muscle fibers from the transverse muscle of the tentacle. The cells at this stage in both the arm and the tentacle are obliquely striated. The tentacle cells at this stage **(C,D)** show mitochondria (M) in the core and rows of tubules of the sarcoplasmic reticulum (S) extending into the center of the cells. Compare with adult ultrastructure shown in Figure 10. The scale bar length equals 1 μm. From Kier (1996).

The ultrastructural differentiation of the transverse muscle of the arms and tentacles of squid is especially relevant for consideration of arm and tentacle regeneration (Kier, 1996). In the loliginid squid *Sepioteuthis lessoniana*, during the first 2 weeks after hatching the tentacle transverse muscle fibers lack the adult ultrastructure and are indistinguishable from the obliquely striated fibers of the transverse muscle of the arms (Figure 11). Transverse striation of the tentacle muscle cells appears at approximately 3 weeks and the adult ultrastructure is present 4–5 weeks after hatching. High speed video recordings of prey capture show correlated behavioral changes. During the first 2–3 weeks after hatching, *Sepioteuthis lessoniana* hatchlings exhibit a different prey capture behavior from the adults that involves a rapid jet forward and capture of the prey with splayed arms. It is not until 4–5 weeks after hatching that the rapid tentacular strike is employed (Kier, 1996). It is unknown if a similar sequence of differentiation occurs during regeneration of the tentacles.

### Ultrastructure of the longitudinal muscles of the arms and tentacles of coleoids

The longitudinal muscle fibers of the arms and tentacles have not been studied in detail with electron microscopy. In previous work on the transverse muscle, the longitudinal muscle bundles are often included in sections so basic observations of their structure have been made. In the arms and in the tentacles they appear to be obliquely striated muscle fibers with ultrastructural characteristics that are similar to those of the transverse muscle of the arms described above (Kier, 1985).

## Contractile properties of the transverse muscle of the arms and tentacles of squid

As described above, the muscle fibers of the transverse muscle of the arms provide support for the relatively slow bending movements while those of the tentacle are responsible for extremely rapid elongation during the prey strike. Although their gross arrangement appears quite similar (compare Figures 1, 4), their function is dramatically different and their contractile properties reflect this difference. In order to characterize their contractile properties, Kier and Curtin (2002) dissected small bundles of fibers from the transverse muscle mass of the arms and the tentacles of *D. pealeii*, which were tested in a muscle mechanics rig equipped with a muscle lever system and force transducer. The length-force relationship, force-velocity relationship and stimulus frequency-force relationship were determined for both the tentacle and the arms fibers.

**Figure 12:**
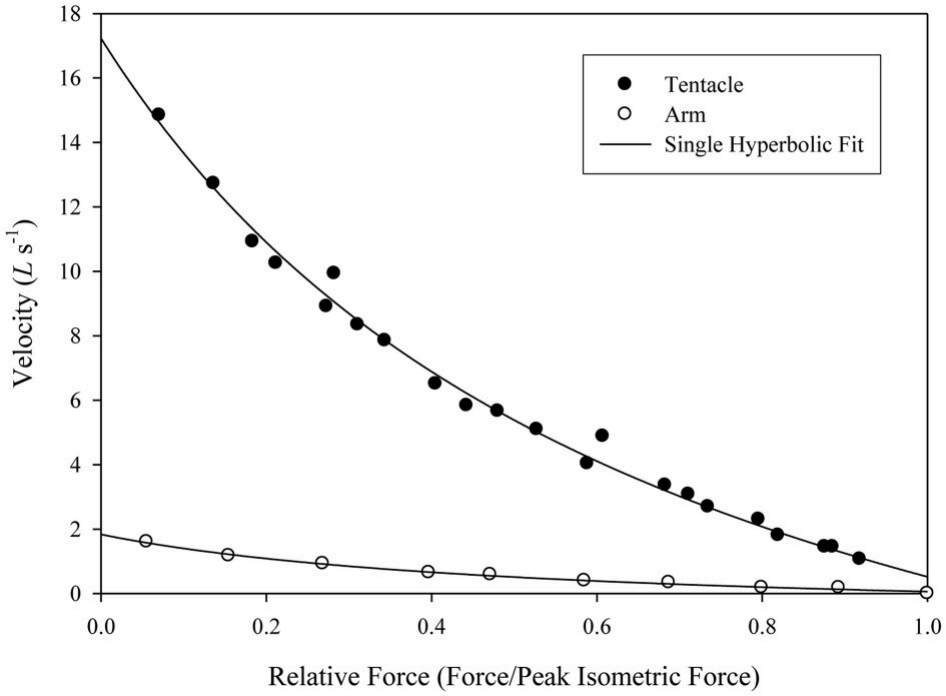
**Force/velocity relationship for a single tentacle transverse muscle bundle preparation (filled circles) and single arm transverse muscle bundle preparation (open circles)**. Force is expressed relative to the isometric force of the preparation (mean of repeat twitches for the tentacle and repeat 100 ms, 50 Hz tetani for the arm, *Doryteuthis pealeii*). Velocity is expressed in *L*_0_ s^−1^, where *L*_0_ is the length of the preparation at which peak isometric force is produced. The lines were fitted to the data using Hill's single hyperbolic function. From Kier and Curtin (2002).

The force-velocity relationship of the two fibers was dramatically different. At 19°C the maximum unloaded shortening velocity of the tentacle transverse muscle fibers was estimated to be as high as 17 lengths per second (mean = 15.4 *L*_0_s^−1^, *SD* = 1.0 *L*_0_s^−1^) while that of the arm transverse muscle fibers was 1.8 lengths per second (mean = 1.5 *L*_0_s^−1^, *SD* = 0.2 *L*_0_s^−1^; Kier and Curtin, 2002; Figure 12). A significant difference in the response to electrical stimulation was also observed. The twitch:tetanus ratio, which is simply the ratio of twitch force to peak tetanic force, was 0.66 (*SD* = 0.06) in the tentacles, but only 0.03 (*SD* = 0.02) in the arms. A significant difference in peak tetanic tension was also observed: using 200 ms tetani, the tentacle fibers generated an average of 131 mN mm^−2^ (*SD* = 56 mN mm^−2^, stimulus frequency at max of 80 Hz) while those of the arm produced a mean of 468 mN mm^−2^ (*SD* = 91 mN mm^−2^, stimulus frequency at max of 160 Hz). Even higher forces, perhaps 25% greater than those measured, could be produced by the arm muscle with longer tetani. The length-force relationship of the arm and the tentacle fibers was found to be similar and no difference was observed in the relationship during twitch vs. tetanic stimulation. High levels of resting tension were observed in both fiber types when they were extended beyond optimal length. The high resting tension appeared to damage the preparations so it was not possible to characterize the descending limb of the length tension curve (Kier and Curtin, 2002). The high resting tension is consistent with a recent study of the mantle muscle of *D. pealeii*, which also incorporated sonomicrometry measurements of the muscle and demonstrated that the mantle muscle fibers operate solely on the ascending limb of the length-tension curve (Thompson et al., 2014).

### Mechanisms responsible for differences in contractile properties of arm and tentacle transverse muscle

The differences in contractile properties between the arm and the tentacle transverse muscle of squid are dramatic, especially with respect to the shortening velocity of the transverse tentacle muscle described above. What specializations of the tentacle muscle fibers are responsible for the high shortening velocity observed?

#### Biochemical comparison of arm and tentacle transverse muscle

**Figure 13:**
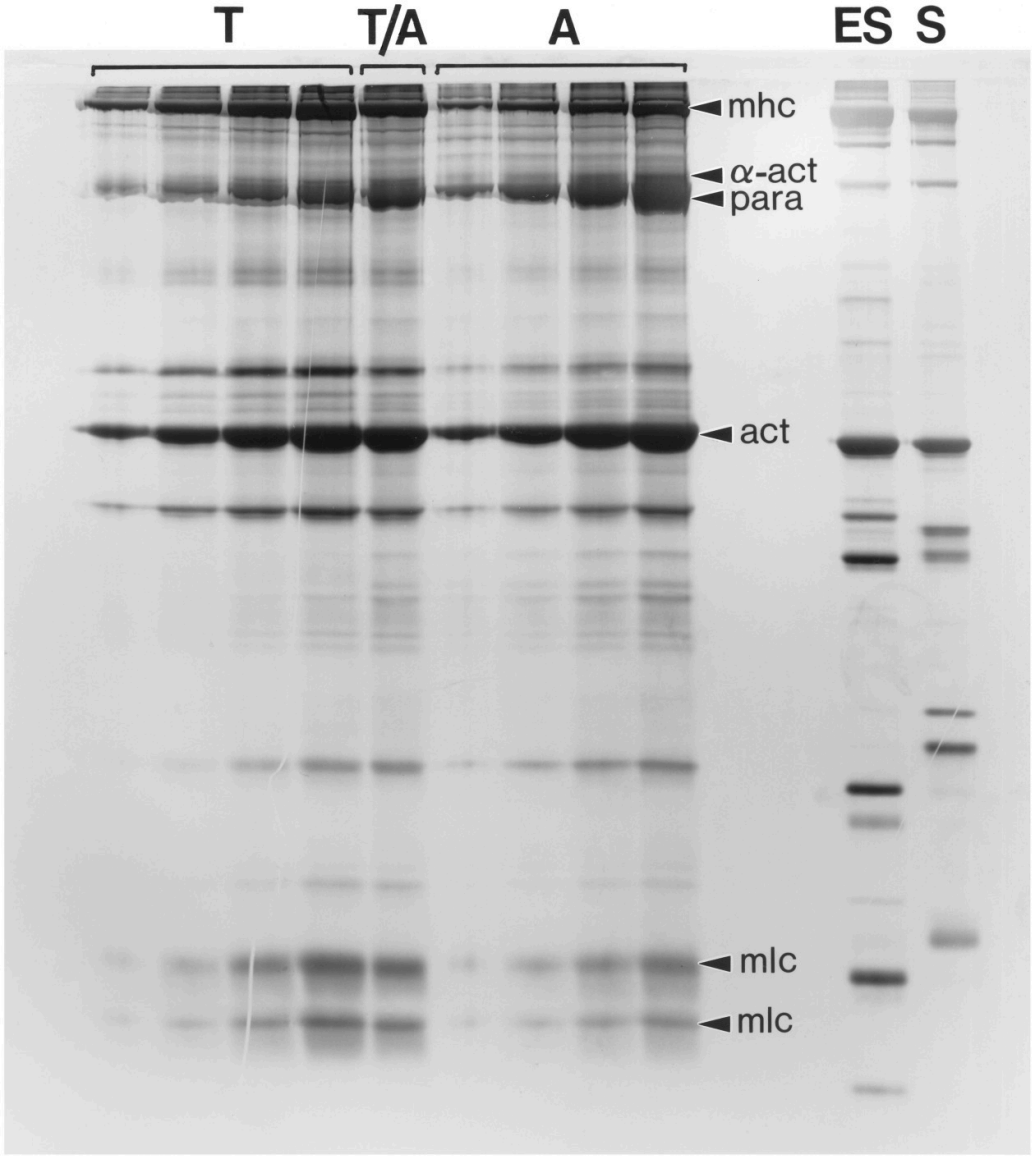
**Photograph of a silver-stained SDS-polyacrylamide gel (10.5% acrylamide) of a range of loadings (increasing from left to right) of myofilament extracts of the transverse muscle cells of the tentacle (T) and arm (A) of *Sepioteuthis lessoniana***. The lane labeled T/A was loaded with 50% tentacle and 50% arm extract. Note that the protein composition of the arm and tentacle transverse muscles is remarkably similar. For comparison, identically prepared myofilament extracts of an erector spinae muscle (ES) (a fast muscle) and a soleus muscle (S) (a slow muscle) from a New Zealand White rabbit were run in adjacent lanes. Several of the bands are identified, including α-actinin (α-act), actin (act), the myosin heavy chains (mhc), the myosin light chains (mlc) and paramyosin (para). From Kier and Schachat (1992).

Kier and Schachat (1992) compared the myofilament protein compositions from the arms and tentacles of the loliginid squid *Sepioteuthis lessoniana* in order to ascertain the possible role of differences in biochemical composition in tuning the contractile properties of these fibers. Samples of myofilament proteins were extracted from the transverse muscle of the arms and the transverse muscle of the tentacles and compared using sodium dodecyl sulfate polyacrylamide electrophoresis (SDS-PAGE; See Kier and Schachat, 1992 for details). Identical techniques have been used to resolve the extensive variety of differences in protein composition in mammalian fiber types, yet very few differences were revealed for the arm and tentacle samples (Figure 13). Of particular relevance for considerations of shortening velocity, no differences in the myosin light chains and myosin heavy chains were observed. In addition, no differences in the myosin heavy chain were resolved using myosin purified from each fiber type and compared using several low percentage gel techniques and also using V8 protease and cyanogen bromide peptide mapping techniques (See Kier and Schachat, 1992) for details. A difference was observed in the content of the thick filament protein paramyosin, which was higher in the obliquely striated arm muscle, and is consistent with previous research showing a correlation between paramyosin content and thick filament length (Levine et al., 1976). Thus, the same techniques that have been employed to document the remarkable biochemical heterogeneity of vertebrate muscle fiber types revealed remarkably few differences in myofilament protein composition between the arm and tentacle fibers, in spite of dramatically different contractile properties.

#### Myosin isoforms in cephalopod muscle

The biochemical techniques describe above are unlikely to resolve highly conserved isoforms of proteins that differ in only a few amino acids. This is relevant to the present discussion because a study (Matulef et al., 1998) that sequenced the myosin heavy chain from the funnel retractor muscle of squid detected two alternatively spliced transcripts from the myosin heavy chain gene. The two alternatively spliced myosin mRNAs for the two isoforms differ in the ATP-binding loop so could potentially impact myosin function and thus muscle fiber contractile properties.

Kier and Schachat (2008) used semi-quantitative RT-PCR with primers that spanned the alternatively spliced region in order to explore the relative abundance of mRNA for the two myosin heavy chain isoforms in the arm transverse muscle and the tentacle transverse muscle of the squid *D. pealeii*. The results revealed low levels of mRNA for the alternatively spliced myosin isoform (termed “B”) in both muscle fiber types with isoform “A” much more abundant (90–95%) in both the tentacle transverse muscle and the arm transverse muscle. Thus, the low levels of the alternatively spliced isoform along with the lack of significant difference in the levels in the tentacle vs. the arm suggest that the difference in shortening velocity is unlikely to be due to differences in expression of myosin heavy chain isoforms.

To resolve the issue of potential differences in the myosin heavy chain composition of the arms and tentacles, Shaffer and Kier (2012) conducted a full analysis of the myosin heavy chain sequence from the transverse muscle of the tentacles and from the transverse muscle of the arms of *D. pealeii*. Transcripts of the myosin heavy chain were sequenced from these muscles and in addition from mantle, fin and funnel retractor musculature. This research showed that the myosin heavy chain was identical in all of the muscles analyzed. Both the A and B isoform described above were found in all muscles, and an additional isoform, isoform “C” was also found in all muscles sampled. In addition to the analysis of squid musculature, Shaffer and Kier (2016) analyzed the myosin heavy chain transcript sequences and expression profiles from the arm, tentacle, mantle, funnel retractor and fin of the cuttlefish *Sepia officinalis* and from the arm, mantle, funnel retractor, and buccal mass musculature of *Octopus bimaculoides*. Four myosin isoforms were identified in *S. officinalis* and six isoforms were identified in *O. bimaculoides*; all isoforms were expressed in all tissue studied. Thus, it appears unlikely that tissue-specific expression of myosin isoforms occurs.

#### Ultrastructural specialization for fast contraction

Specialization of the tentacle transverse muscle fibers for high shortening velocity appears to have involved primarily ultrastructural modifications. Given the lack of biochemical specialization and the lack of evidence for tissue-specific expression of myosin isoforms, the cross-bridge cycling rate and interfilamentary sliding velocity of the arm and tentacle muscle are likely to be similar. As described above, the shortening velocity of the fibers of the transverse tentacle muscle was 10 times greater than that of the transverse arm muscle. This dramatic difference in properties is most likely primarily due to differences in the thick filament lengths of the two fiber types. The thick filaments in the tentacle fibers were found to be one tenth the length of those of the arm fibers and thus the tentacle fibers have ten times as many elements in series, per unit length. Because shortening velocities of elements in series are additive (Huxley and Simmons, 1973; Josephson, 1975), based simply on the relative thick filament and sarcomere proportions, the maximum shortening velocity of the tentacle fibers would be expected to be ten times that of the arm muscle fibers. As described above, this was indeed found to be the case with a mean unloaded shortening velocity for the tentacle fibers of 15 *L*_0_s^−1^ and 1.5 *L*_0_s^−1^ for the arm fibers.

## Summary

The musculature and connective tissue of the arms and tentacles of coleoid cephalopods is arranged in a complex, three-dimensional array. Support and movement in these structures depends on a form of hydrostatic skeletal support, referred to as a muscular hydrostat, in which the musculature serves both for force generation and as the support for movement. Because the muscle and other tissue of the arms and tentacles resist volume change, any decrease in one dimension must result in an increase in another. Since the arms and tentacles possess muscle fibers running in all three dimensions, active control of all dimensions can be achieved, allowing great diversity and complexity of movement, including elongation, shortening, bending and torsion. In addition to deformation and movement, these appendages are also capable of active control of tensile, compressive, bending, and torsional stiffness. The musculature of these appendages is predominantly obliquely striated with relatively long myofilaments. The exception is the transverse muscle of the tentacles of squid and cuttlefish, which is responsible for remarkably rapid elongation during the prey capture strike. This muscle exhibits cross striations and unusually short thick filaments and sarcomeres. Its shortening velocity is an order of magnitude higher than the obliquely striated fibers of the arms, most likely due to these ultrastructural differences since biochemical comparisons reveal remarkable similarity in the proteins of the myofilament lattice and identical myosin heavy chain sequences in the cross-striated and obliquely striated fibers. Additional research on the mechanisms and control of regeneration is of particular interest, given the remarkable complexity of the arrangement of the muscle and connective tissues of these appendages.

## Author contributions

The author confirms being the sole contributor of this work and approved it for publication.

### Conflict of interest statement

The author declares that the research was conducted in the absence of any commercial or financial relationships that could be construed as a potential conflict of interest.
